# Migration protocol to estimate metal exposure from mouthing copper and tin alloy objects

**DOI:** 10.1186/1476-069X-13-66

**Published:** 2014-08-11

**Authors:** Paola Urrestarazu, Germán Villavicencio, Margaret Opazo, José Arbildua, Craig Boreiko, Katrien Delbeke, Patricio H Rodriguez

**Affiliations:** 1 Center of Ecotoxicology and Chemistry of Metals, Universidad Adolfo Ibañez, Diagonal Las Torres, 2700 Peñalolen, Santiago, Chile; 2 International Lead Zinc Research Organization, 1822 NC Highway 54 East, Suite 120, Durham, NC 27713, UK; 3 European copper Institute, Avenue de Tervueren 168 b10, B-1150 Brussels, Belgium

**Keywords:** Lead, Mouthing, Migration test, Alloys, Chronic exposure, Saliva

## Abstract

**Background:**

Low blood lead levels previously thought to pose no health risks may have an adverse impact on the cognitive development of children. This concern has given rise to new regulatory restrictions upon lead metal containing products intended for child use. However few reliable experimental testing methods to estimate exposure levels from these materials are available.

**Methods:**

The present work describes a migration test using a mimetic saliva fluid to estimate the chronic exposure of children to metals such as lead while mouthing metallic objects. The surrogate saliva medium was composed of: 150 mM NaCl, 0.16% porcine Mucin and 5 mM buffer MOPS, adjusted to pH 7.2. Alloys samples, in the form of polished metallic disc of known surface area, were subjected to an eight hours test.

**Results:**

Two whitemetal alloys Sn/Pb/Sb/Cu and three brass alloys Cu/Zn/Pb were tested using the saliva migration protocol. In the case of the whitemetal alloys, first order release kinetics resulting in the release of 0.03 and 0.51 μg lead/cm^2^ after 8 hours of tests were observed, for lead contents of 0.05-0.07% and 5.5%, respectively. Brasses exhibited linear incremental release rates of 0.043, 0.175 and 0.243 μg lead/cm^2^h for lead contents of 0.1-0.2%, 1.7-2.2% and 3.1-3.5%, respectively. The linear regression analysis of lead release rates relative to Pb content in brasses yielded a slope of 0.08 μg lead/cm^2^h%Pb (r^2^ = 0.92). Lead release rates were used to estimate the mean daily mouthing exposure of a child to lead, according to age-specific estimates of mouthing time behavior. Calculated daily intakes were used as oral inputs for the IEUBK toxicokinetic model, predicting only marginal changes in blood lead levels (0.2 μg lead/dL or less) for children aged 0.5 to 1 years old exposed to either class of alloy.

**Conclusions:**

The results of this study as a whole support the use of migration data of metal ions, rather than total metal content, to estimate health risk from exposure to metals and metal alloys substances in children.

## Background

Metals are part of our everyday life and can be found in clothing, sanitary tap water, jewelry, electronic devices, vehicles and infrastructure in general. They are also present in drinking water, food and the air we breathe as result of both natural and anthropogenic sources. Therefore humans are simultaneously exposed to metals from different pathways: inhalation, dermal contact and ingestion. The consequences of this exposure for human health vary as a function of exposure intensity and the specific metal in question. On one hand, some metals (e.g. sodium, potassium, calcium, magnesium, iron, copper, zinc, selenium, chromium and molybdenum) are essential for life and are normally absorbed from water and food, although above certain exposure levels they may be harmful. On the other hand, humans are also exposed to non essential metals that can be absorbed using passive mechanisms or by competition using active transport mechanisms intended for essential metals [1,2]. An example of the complex relationship between essentiality and toxicity of metals has been thoroughly studied for copper, where a deficit of the metal has been related to hypochromic anemia, neutropenia, hypopigmentation of hair and skin, bone fragility, vascular abnormalities and detriment in brain functions [1]. On the other hand an excess of oral copper intake may cause intravascular hemolysis, liver cirrhosis, hypotension, tachycardia and acute renal failure [1].

Exposure to non-essential metals, and lead in particular, is in general a greater concern due to the widespread presence of this metal in consumer goods. Lead is a cumulative toxicant that may produce neurological, hematological, gastrointestinal, cardiovascular and renal effects [3]. The metal uptake occurs through passive diffusion in the gastrointestinal tract and by competing with essential metals for their active transport mechanisms. Lead absorption is highly variable as a function of factors such as the chemical form in which the metal is ingested, the age of the individual, the food type and fasting status. Children are particularly sensitive to lead because their gastrointestinal absorption is higher and their nervous systems are not fully developed [3]. Thus, a major public health concern over lead exposure comes from studies that relate low levels of lead in blood to decrements in IQ [4,5].

Public exposure to lead has been declining due to the elimination of lead alkylated additives (anti-knocking agents) in gasoline and new regulations limiting the use of lead and lead compounds in paints, lead soldered food cans, potable water systems etc. [6,7]. For example, U.S. Consumer Product Safety Commission bans paints or other similar surface coatings, if their total lead content is above 0.009%, in articles intended for use by children (Code of Federal Regulations (CFR), Title 16, Part 1303). European regulation instead, is based on lead bioavailability from toys materials, considering three categories (type of material) with migration limits, as tested by EN 71–3:2013 [8], between 3.4 and 160 mg/kg. The declines in general population blood lead levels have been accompanied by demonstrations that low levels of lead exposure previously thought to pose no risk may indeed have adverse impacts upon the cognitive development of children. This has placed emphasis upon the evaluation of lead exposure routes and sources previously thought to have little consequence.

Due to the ethical and health considerations associated with *in vivo* studies to determine metal exposure or metal bioavailability in humans, surrogate studies that measure the release of substances in physiological mimetic fluids have been developed. These studies have generated the concept of bioaccessibility which is defined as the fraction of a substance that is available for absorption when placed into *in vitro* surrogates for body fluids. Bioaccessibility studies using a wide range of surrogates for human fluids have provided exposure information for use in assessing the hazard characteristics or risks of chemicals. The tests include approximations to estimate exposure in a particular body compartment, such as: interstitial, alveolar, lysosomal, synovial, gastric and intestinal fluids [8-12].

In the particular case of mouthing behavior mediated exposure in children, where no ingestion of the article occurs, several *in vitro* studies to estimate exposure in a variety of artificial saliva fluids have been described in the literature [13-16]. The described methodologies were used to assess the migration of di-(2-ethylhexyl) phthalate (DEHP) and di-isononyl phthalate (DINP) from PVC articles. Since PVC is a soft material, in general two mouthing exposure scenarios were considered, one that mimicked mainly sucking events and a second one that included chewing, usually simulated by the presence of marbles or the repetitive impact of a pneumatic piston on the PVC sample submerged in artificial saliva fluid. However, none of these methods considers the scenario of mouthing exposure of a metallic article for a certain amount of time in which, due to its hardness, would entail mainly licking and sucking behaviors.

The main goal of the present work was to develop a reliable *in vitro* method to estimate metal bio accessibility from metallic samples in a massive form (10 cm^2^ exposed surface) in a medium that mimics saliva in order to estimate metal release that might be associated with metallic articles mouthed by children.

## Methods

### Reagents

Pig gastric Mucin was obtained from Aldrich M2378 (Mucin type II from pig stomach), containing approximately 1% sialic acid and bovine sub maxillary gland Mucin was obtained from Worthington Biochemical Corporation LS002976, containing between 9-17% of sialic acid. The reagents used to prepare the saliva medium were Merck pro analysis grade. The reagents used to perform the analytical measurements were Merck Suprapur quality (low in trace metals).

### Alloys

#### Tin alloys and lead

Samples of whitemetal alloys used for jewellery and model casting were obtained from Fenix Metals, UK Branch. The samples were identified as: Pure lead, Tin/Lead Alloy 3 and Tin/Lead Alloy 5 (samples description in Table 1). Three disc samples of each substance were used.

**Table 1:** Samples description

**Sample**	**Disc diameter**	**Composition (w/w)%**
Pure Lead	4.84 cm	Pb 99.97%
Tin/Lead Alloy 3	4.80 cm	Sn 90.9%; Sb 7.5%; Cu 1.5%; Pb 0.05-0.07%
Tin/Lead Alloy 5	4.82 cm	Sn 91%; Sb 3.5%; Pb 5.5%
M57 (CW510L)	3.9 cm	Zn 40.5-42.7%; Cu 57-59%; Pb 0.1-0.2%
Z45 (CW602N)	4.1 cm	Zn 34.7-36%; Cu 62.3-63%; Pb 1.7-2.2%; As 0.04-0.08%
Z33 (CW614N)	4.6 cm	Zn 38.3-39.6%; Cu 57.2-58.2%; Pb 3.1-3.5%

#### Copper alloys

Samples were obtained from Wieland-Werke AG, Ulm, Germany. The samples were identified as Brass CuZn42 (CW510L) label “M57”, Brass CuZn36Pb2As (CW602N) label “Z45” and Brass CuZn39Pb3 (CW614N) label “Z33” (samples description in Table 1). Three disc samples of each alloy were used.

### Polishing procedure

**Figure 1:**
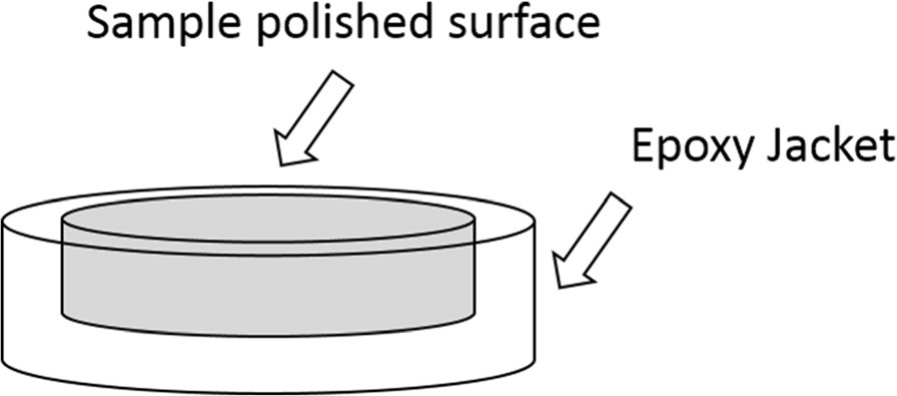
**Schema of an alloy sample.** Samples were mounted in epoxy resin with upper face polished and exposed to the medium.

Samples were cold mounted in epoxy resin (Epofix from Struers Inc, Denmark) to avoid metal abrasion against the vessel walls, and one face was left exposed in order to carry out the migration tests (Figure 1).

Mounted samples were polished to decrease surface heterogeneity due to corrosion and passivation processes, and to assure a uniform defined surface condition at the beginning of the migration test. The procedure included wet grinding the samples with SiC papers of 320, 600, 1200 and 2000 grit, for one minute each. Only gentle pressure was applied during grinding to avoid a memory imprint of the scratches. After being wet ground, the samples were rinsed in ethanol and dried with nitrogen gas to avoid corrosion during sample preparation. Polishing was performed using 3, 1 and 0.25 μm diamond paste. To obtain scratch-free surfaces, the samples were held very gently against the rotating polishing cloth and ethanol in excess was used as lubricant. Each polishing step with diamond paste on cloth took 5 to 10 minutes, depending on the presence of scratches. If the sample presented small spots on the surface at the end of the process, as observed under an optical microscope, the polishing steps was repeated. To remove any residual particles left on the surface after polishing, all the samples were cleaned ultrasonically, first in acetone for 6 minutes and then in isopropyl alcohol for another 6 min. All the samples were quickly dried in a cold stream of nitrogen gas.

In the case of pure lead samples the polishing protocol was modified to avoid SiC and diamond particle inclusions in the soft metallic surface of lead during the polishing procedure with abrasives. Shorter polishing steps, alternated with etching steps of 5 seconds in a 75% (v/v) acetic acid (glacial) and 25% hydrogen peroxide (100 volumes) mix, were performed in order to remove attached abrasive particles.All the samples were stored in a desiccator for 24 hours before the beginning of the test. A scheme of the mounted sample is presented in Figure 1.

### Saliva migration test

Metals or metal alloys samples mounted in epoxy resin and polished according to the protocol described previously, were prepared. The resulting samples, with a total exposed surface between 10 and 20 cm^2^, were submitted to the migration test in 0.5 L of synthetic saliva medium, composed of Mucin 0.16%, NaCl 150 mM and MOPS 5 mM at pH 7.2. The synthetic medium was based upon US CPSC [14,17], artificial saliva but NaCl 150 mM (isotonic solution) and MOPS (no metal binding buffer) were added to replace the PBS buffer in order to prevent precipitation of metal phosphate complexes. Artificial saliva medium was freshly prepared before each test.

Sample testing and blanks were performed in triplicate using HDPE flasks with 0.5 L of synthetic saliva medium. The flasks with the medium were pre incubated in an orbital shaker at 60 RPM, until equilibrium temperature was reached (37°C); pH and dissolved oxygen were measured. The test began when the mounted samples were added to the saliva medium. Aliquots of 10 ml were taken from samples and blanks (by triplicate) at time intervals of: 0, 1, 2, 4 and 8 hours. After each sampling time, an equal amount of fresh medium was added back, in order to keep the ratio exposed surface area to solvent volume constant. To preserve sample integrity after collection, 2 drops of concentrated nitric acid (Merck, Suprapur) was added. After this step, denatured protein was precipitated by centrifugation. The sample tubes (polypropylene, 15 ml) were stored for 1 hour at 4°C before centrifugation (3400 g × 10 min). The resulting supernatant was then passed through a 0.45 μm PVDF membranes and stored at 4°C.

Dissolved metal concentrations were measured using an ICP-MS Perkin Elmer model ELAN 9000 system, located in a cleanroom, where the incoming air is filtered to keep a low level of airborne particles. In this particular case, the cleanroom meets the standard requirement for class 10,000 [18]. Metals detection limit were calculated from the blank samples measurements of each test, with values of: 18 μg/L for zinc, 1 μg/L for tin, between 0.2 and 0.5 μg/L for lead and between 3 and 7 μg/L for copper. Quality assurance and quality control measurements were performed every ten samples, and included: a duplicate reading, randomly chosen from the previous 10 samples (to assess instrument stability); a sample spiked with a standard (ICP-MSCS from High Purity Standards Inc) to determine possible analyte signal interference due to the high concentration of other matrix constituent (matrix effect); and two samples composed of independent multi metallic standards (QCS-26 and QCS-27, from High Purity Standards Inc, Charleston, South Carolina, USA), diluted in saliva medium.

## Results and discussion

### Saliva migration test

A migration method of saliva extraction was designed to mimic chronic mouthing of metallic objects. The method is based on US CPSC 1998 [17], but modifications were adopted to avoid interactions between medium components and metallic ions (i.e. precipitation) such as the use of MOPS buffer and NaCl instead of PBS buffer.

#### Effect of the degree of glycosylation of Mucin protein in metal release

The existence of different iso species of Mucin protein, a main component of saliva fluid, raised the question of the appropriate Mucin to be used as part of the synthetic media to test metallic compounds. Sub maxillary protein has more regions rich in serine/threonine amino acids than stomach protein and therefore the degrees of glycosylation of the purified proteins are quite different, 1% in the stomach protein and 9-17% in the sub maxillary gland protein. To test the role of the protein source in the saliva migration protocol, a comparative *in vitro* assay of whitemetal alloys samples with low (0.05 – 0.07%) and high (5.5%) lead content, respectively, was performed. Metallic disks embedded in epoxy resin and with one face exposed to the media, were assayed for 8 hours in the two different saliva mimetic fluids. The composition of the first medium contains 0.16% of Mucin from pig stomach, and the second medium contains 0.16% of Mucin from bovine sub maxillary gland.

**Figure 2:**
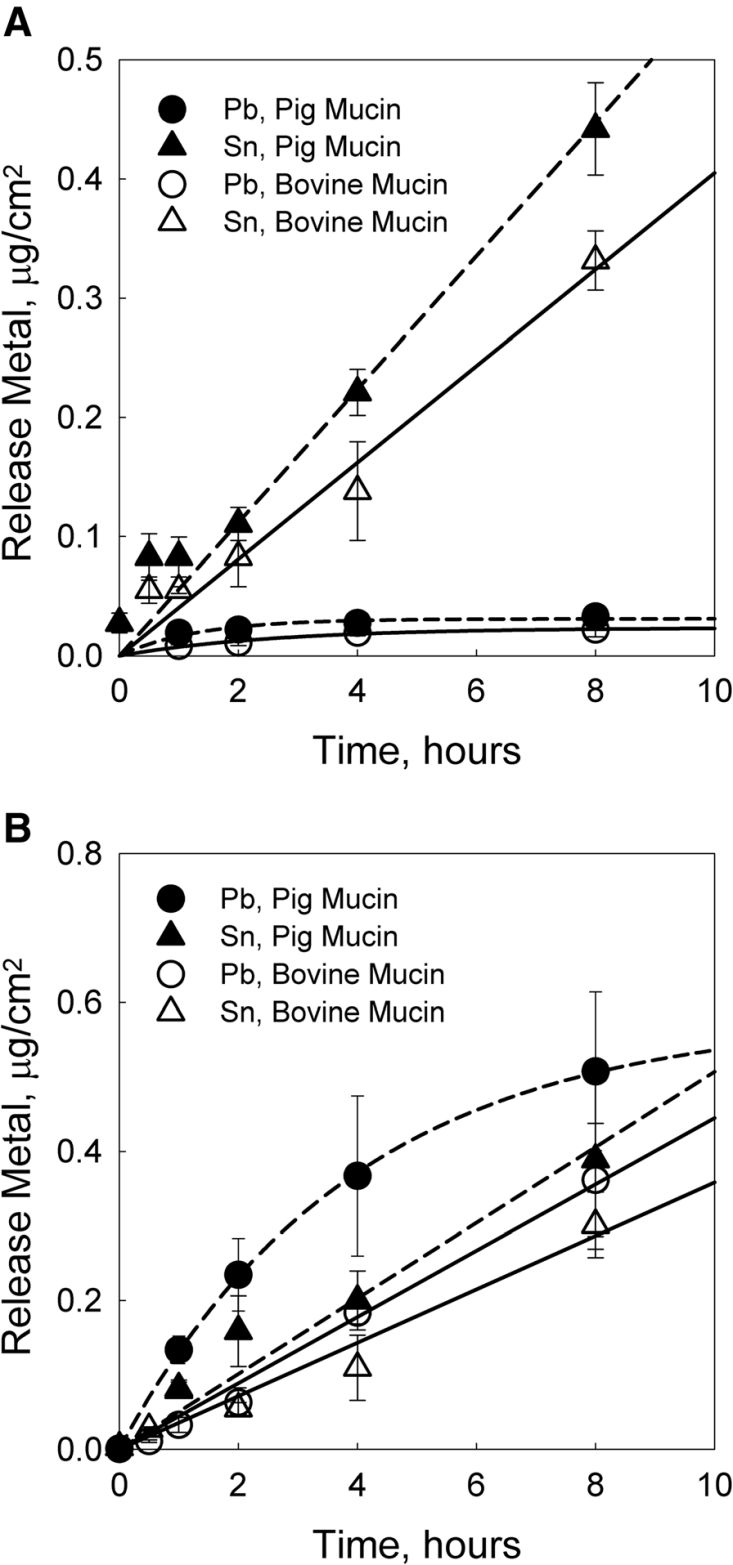
**Time course of the metal released from whitemetal alloys.** Two parallel migration tests were carried out for 8 hours, one in synthetic saliva medium with stomach Mucin species and the other in saliva with sub maxillary gland Mucin specie. Tests were performed at pH 7.2, 37°C and with 60 rpm agitation rate. Plot **A** shows metal release kinetics of tin/lead alloy 3 sample in both media, and plot **B** the metal release kinetics of tin/lead alloy 5 sample in both media. Lead and tin released in saliva with stomach Mucin are presented in filled circle and triangle symbols, respectively; and lead and tin released in saliva with sub maxillary gland Mucin are presented in hollow circle and triangle, respectively. Dashed lines correspond to linear or first order kinetic fits of the results obtained with stomach Mucin species (pig); solid lines correspond to linear or first order kinetic fits of the results obtained with sub maxillary gland Mucin (bovine).

The tin/lead alloy samples tested with pig Mucin yielded releases of 0.44 ± 0.04 and 0.39 ± 0.12 μg/cm^2^ for tin, and of 0.03 ± 0.006 and 0.51 ± 0.11 μg/cm^2^ for lead (for Pb contents of respectively <0.1% and 5.5%) after 8 hours of incubation. On the other hand, the bovine Mucin tests gave releases of 0.33 ± 0.02 and 0.30 ± 0.04 μg/cm^2^ for tin, and of 0.02 ± 0.006 and 0.36 ± 0.076 μg/cm^2^ for lead (for Pb contents of respectively <0.1% and 5.5%). No copper release above the method detection limit was detected in any of the tin/lead alloys samples submitted to the saliva migration tests. The results of metal release for alloys samples 3 and 5 in both media are presented in Figure 2.The lead releases from alloy sample 3 (<0.1% Pb) were fit to a first order kinetic function, rising to a maximum value around 8 hours. The tin release for the same sample showed a linear increment; therefore a linear regression through the origin was fitted. Higher lead content in alloy sample 5 (5.5% lead) correlated with the observed metal release after 8 hours of incubation in Figure 2B, and with the lead release values reported in the previous paragraph. Only the lead release in the pig Mucin tests exhibited a clear first order kinetic function. The lead release from tin/lead alloy sample 5 in the bovine Mucin tests increased linearly during 8 hours of incubation. In general, similar metal releases were found for tin/lead alloy 3 and 5 samples in the comparative saliva tests, with a tendency to higher metal releases in the case of the pig Mucin test (p < 0.05, for tin and lead). Mucin from pig stomach was considered as suitable replacement for the sub maxillary gland protein, because it represented a worst case scenario for metal bioaccessibility.

#### Metal recovery

A two hours migration test in saliva medium using pig stomach Mucin and a multi-metallic spike of 100 μg/L (final concentration) was performed. Recoveries above 93% were obtained for 8 different metals, including: As, Cd, Co, Cu, Mo, Ni, Pb and Zn. The recovery results, shown in Table 2, exclude possible metal losses associated with co-precipitation with Mucin, metal binding to volumetric flasks or filters and metal interactions with the matrix to produce insoluble species.

**Table 2:** Metals recovery (%) assays in saliva medium with pig stomach Mucin

	**Vessel 1**				**Vessel 2**				**Vessel 3**						
**Metal**	**R1**	**R2**	**R3**	**CV1, %**	**R1**	**R2**	**R3**	**CV2, %**	**R1**	**R2**	**R3**	**CV3, %**	**Mean**	**St.Dev.**	**CV, %**
**Zn**	100	94	106	6	102	103	102	1	98	102	111	6	**102**	**4.7**	**5**
**Co**	97	97	101	2	101	101	102	0	98	101	101	2	**100**	**2.0**	**2**
**Ni**	99	99	104	3	103	103	104	1	101	103	103	1	**102**	**1.8**	**2**
**Pb**	87	89	95	4	95	95	95	0	94	95	97	1	**93**	**3.1**	**3**
**As**	99	95	101	3	100	100	101	0	97	99	102	2	**99**	**2.0**	**2**
**Mo**	92	94	96	2	97	98	99	1	95	95	97	1	**96**	**2.1**	**2**
**Cd**	99	99	104	3	102	102	102	0	99	102	102	2	**101**	**1.8**	**2**
**Cu**	97	96	101	3	101	101	102	1	99	101	103	2	**100**	**2.3**	**2**

CV1, CV2 and CV3 correspond to the coefficient of variation within each vessel (vessels 1, 2 and 3). CVs correspond to the ratio between the standard deviation of each set of R1, R2 and R3, and the average of the same 3 values (as percentage).

CV corresponds to the coefficient of variation between vessels.

R1, R2, R3 correspond to the measurements performed to the three replicas in each vessel.

St.Dev.: Standard Deviation.

#### Metal release from brass alloys in saliva migration test

**Figure 3:**
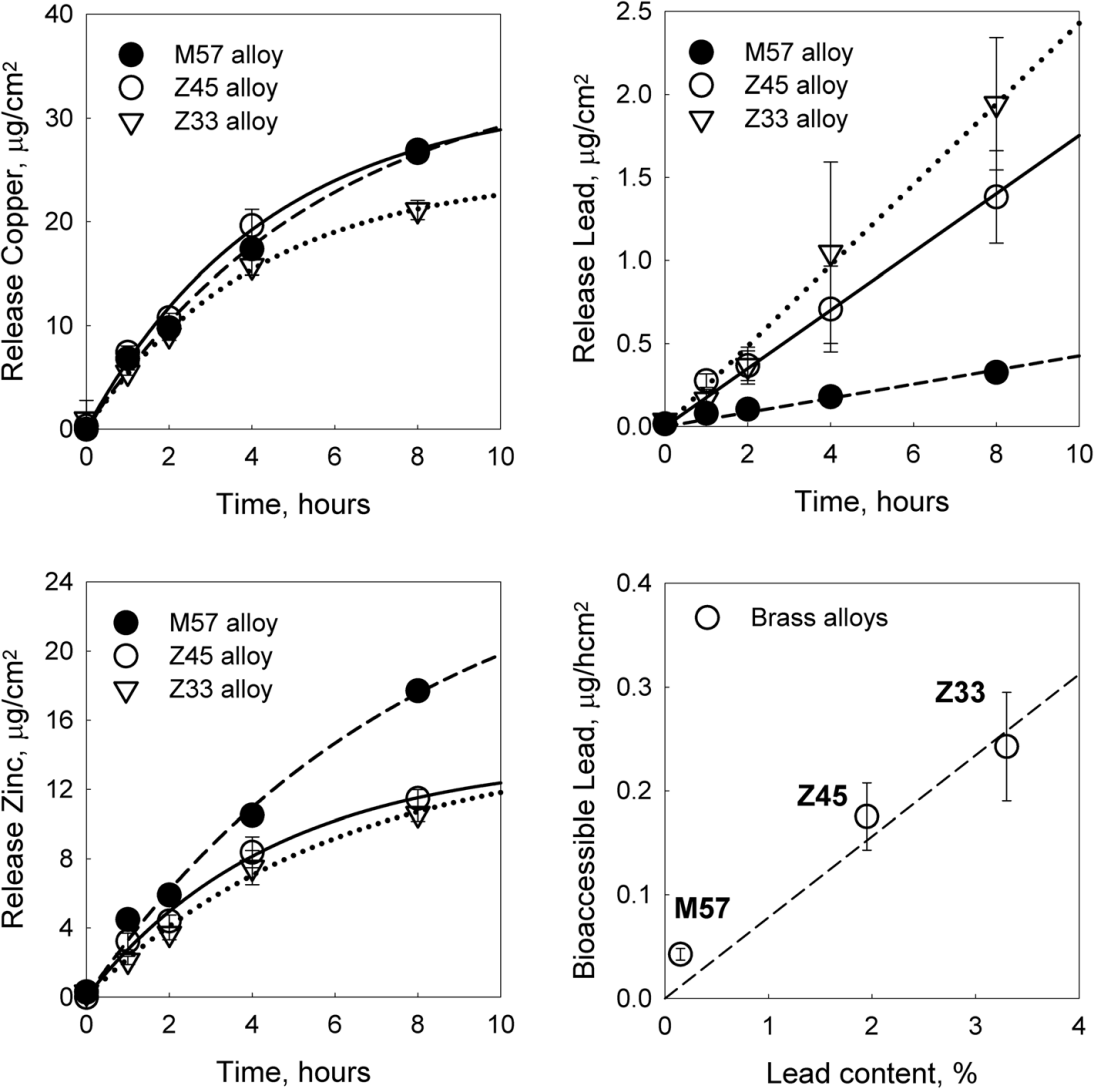
**Time course of metals released from brass alloys.** Migration tests of brasses M57 (Pb 0.1-0.2%), Z45 (Pb 1.7-2.2%) and Z33 (Pb 3.1-3.5%) were carried out for 8 hours in synthetic saliva medium with stomach Mucin specie at pH 7.2, 37°C and with 60 rpm agitation rate. Results of copper, lead and zinc measurements at time 0, 1, 2, 4 and 8 hours of incubation are represented in black circles for M57, white circles for Z45 and white triangles for Z33. Linear (zero order) or first order kinetic fits for each set of data are represented in dashed lines for M57, solid lines for Z45 and dotted lines for Z33. Linear regression for lead release, reported slope values of 0.043 ± 0.006, 0.175 ± 0.035 and 0.243 ± 0.051 μg/hcm^2^ for alloys M57, Z45 and Z33, respectively. Bottom right plot, presents the positive correlation between metal release in μg/hcm^2^ and lead content (% w/w) in the brass samples; dashed line corresponds to the linear regression through the origin, resulting in a slope of 0.08 μg/hcm^2^ %Pb (r^2^ = 0.92).

Brass alloys with different lead content were evaluated for metal release using the saliva extraction protocol. Three alloys were analyzed: M57 with a lead content between 0.1 and 0.2%, Z45 with a lead content between 1.7 and 2.2%, and Z33 with a lead content between 3.1 and 3.5%. Metals released at the end of the 8 hours tests, included copper, with similar releases for alloys M57 and Z45, (26.7 ± 1.0) and (26.9 ± 0.9 μg/cm^2^), respectively, after eight hours of incubation. Lower releases of copper were found for Z33, 21.1 ± 0.9 μg/cm^2^. Zinc release levels for M57, Z45 and Z33 were 17.7 ± 0.3 μg/cm^2^, 11.5 ± 0.5 μg/cm^2^ and 10.6 ± 0.4 μg/cm^2^, respectively after eight hours of incubation. Lead release exhibited a linear increment with time for all the brass samples; therefore a linear regression through the origin was adjusted to each data set (Figure 3). The slopes of the regressions are provided in Figure 3. Copper and zinc presented first order kinetic metal release behavior with time, reaching a maximum metal release level at times longer than 8 hours (Figure 3). A positive correlation between lead release in the saliva migration test and total lead content in the alloys is presented in the bottom right plot of Figure 3, with an overall average lead release from brasses of 0.08 μg/hcm^2^ %Pb (r^2^ = 0.92).

A pure massive lead sample was also evaluated for metal release in synthetic saliva medium (results not shown), where a rate of 25 ± 3 μg/hcm^2^ was calculated from the slope of the linear regression; a much higher release than the one expected from whitemetal alloy or brass alloys with high lead content.

### Estimated blood lead concentrations from the Integrated Exposure Uptake Biokinetic Model (IEUBK) for lead in children

The IEUBK toxicokinetic model (for windows v1.1, build 11) is an exposure assessment tool used to estimate average blood lead concentrations in young children exposed to lead from different sources (air, soil/dust, diet and water) via ingestion or inhalation. The model estimates the continuous (daily) uptake of lead over time with age-dependent adjustments (0–7 years old) being made to basic physiological parameters that modulate the concentration of lead in blood. Age adjustments are also made to the intake rates of the various environmental media that can contain lead. The model default values for lead in different environmental media represent average exposure values for the United States in the general time frame of 1995–2003 [19,20]. The potential significance of lead release during saliva migration tests was evaluated using simple model simulations that compared baseline blood lead estimates using the model default values with those in which oral lead intake were increased by an amount that might result from mouthing activity. This additional lead exposure was entered into the model using the model’s “Alternate Source Data” option, in which the daily lead ingestion value can be increased and associated with an appropriate absorption rate. In the present simulations an absorption rate of 50% was used to estimate the impact of lead extracted in saliva, an uptake rate equivalent to that used to model the uptake of lead in drinking water.

Predicted lead concentrations in blood for an average child were modelled using lead intakes estimated from the mean daily mouthing time of consumer products reported in Greene 2002 [21] (Table 3). The most suitable data for the present study correspond to mouthing time of “other objects” (excluding fingers, toys, dummies/pacifiers and teethers), because no direct observation of mouthing of metallic objects was recorded and would be expected to be scarce due to child selectivity [14]. This data was gathered according to children’s age groups, due to the age-dependent changes in mouthing behavior during growth. In addition to the prediction of the blood lead concentration resulting from average mouthing time estimates, model predictions were also generated using the upper 95th percentile estimate of mouthing time by a child.

**Table 3:** Daily mouthing habits in children

**Age group (months)**	**Estimated mean daily mouthing time for “other objects” (minute: second)**			
	**Norris & Smith **[22]	Groot et al.* [23]	Greene** [21]	**Juberg et al. **[24]
1-3	05:14	–	–	09:00
3-6	12:29	03:00	22:30	09:00
6-9	24:30	09:00	22:30	09:00
9-12	16:25	09:00	22:30	09:00
12-15	12:02	07:00	21:00	09:00
15-18	23:01	07:00	21:00	09:00
18-21	19:49	02:00	21:00	02:00
21-24	12:53	02:00	21:00	02:00
24-36	21:46	02:00	18:42	02:00
36-48	15:16	–	–	–
48-60	10:44	–	–	–
60-72	10:00	–	–	–

*Values were extracted from Figure 1 of Steenbekkers [25] where the original study data (Groot et al. [23]) was analyzed.

**Reported values of minutes per hour were transformed to daily mouthing time, multiplying the hour based time by the number of hours awaken of the children in each age group: 3–12 month - 9 hours awake, 12–24 month - 10 hours awake, and 24–36 month - 11 hours awake (taken from Norris B. & S. Smith, [22]).

Lead exposure estimates were derived assuming mouthing of a 10 cm^2^ metal surface on a daily basis [6,15]. The quantity of predicted lead release during mouthing was estimated from the time course data for lead release in the saliva extraction protocols. Mean estimated mouthing times of 22:30 minutes/day, 21:00 minutes/day and 18:42 minutes/day were used for the age groups of 0.5 to 1, 1 to 2 and 2 to 3 years old, respectively. A mean daily mouthing time of 22:30 minutes/day may overestimate exposure given that a mean “other object” mouthing time of 9 minutes/day has been estimated by Groot et al. and Juberg et al. [23,24] for the same age range, but was selected as the conservative approach to yield worst case exposure estimates.

IEUBK estimates of blood lead concentration using default exposure values are 3 μg/dL for a hypothetical child of 0.5 to 1 years of age, 3.5 μg/dL between 1 and 2 years of age and 3.2 μg/dL between 2 and 3 years of age. Table 4 shows the model predictions for increases in blood lead concentrations after adding the effect of mouthing each particular alloy for both the mean daily mouthing time and 95th percentile of the mouthing time distribution. The net predicted contribution of hypothetical alloy mouthing to the blood lead concentrations of the different age groups is presented in columns labeled IEUBK of Table 4. Predicted blood lead increases up to 0.2 μg/dL were estimated for the alloys when the mean daily mouthing exposure time per age group was used. If the 95th percentile of the time exposure distribution was considered, a maximum net contribution of 0.7 μg/dL for Z33 brass sample was estimated. Release from the metallic lead sample was predicted to markedly increase blood lead levels - an incremental increase of 10.2 μg/dL was predicted when the mean daily mouthing exposure time was used, and 26.7 μg/dL when the upper 95% percentile mouthing time estimate was used (children of 2–3 years of age).

**Table 4:** IEUBK predicted increase in blood lead concentration due to alloys mouthing exposure in children

**Mouthing exposure daily time**	**Alloy**	Calculated daily lead intakes (μg) by mouthing exposure and IEUBK net blood lead contribution (μg/dL)* per age group					
		**0.5–1 years**		**1–2 years**		**2–3 years**	
		**Intake**	**IEUBK**	**Intake**	**IEUBK**	**Intake**	**IEUBK**
Mean	Brass Z33	0.910	0.2	0.849	0.1	0.757	0.1
	Brass Z45	0.658	0.2	0.614	0.1	0.547	0.1
	Brass M57	0.160	0.1	0.149	0.0	0.133	0.0
	Tin/lead alloy 3	0.077	0.1	0.072	0.0	0.065	0.0
	Tin/lead alloy 5	0.530	0.2	0.496	0.1	0.445	0.1
	Pure Lead	93.75	15.0	87.50	12.5	78.00	10.2
95th Percentile	Brass Z33	2.840	0.7	2.670	0.5	3.155	0.5
	Brass Z45	2.052	0.5	1.929	0.3	2.280	0.4
	Brass M57	0.498	0.2	0.469	0.1	0.554	0.1
	Tin/lead alloy 3	0.183	0.1	0.176	0.0	0.195	0.0
	Tin/lead alloy 5	1.500	0.4	1.422	0.2	1.641	0.3
	Pure Lead	292.5	30.8	275.0	27.1	325.0	26.7

^*^IEUBK predicted values minus the baseline lead contribution of default exposure pathways.

The estimates of metal migration and incremental blood lead increases lack *in vivo* validation due to the obvious inability to perform a controlled study in children. To validate similar methodologies for plasticizers in flexible vinyl products [13,15] a comparison against concentration of the substance of interest in the saliva of adult human volunteers was performed, introducing additional uncertainties related to children versus adult mouthing behavior. However, the current test is expected to represent a conservative estimation of mouthing behavior in children due to the nature of the metallic objects, particularly their hardness that limits interaction with the mouth of a child.

## Conclusions

Metal release from lead containing alloys due to mouthing activity in children was estimated using a migration protocol in synthetic saliva medium. Alloy samples were embedded in an epoxy jacket to avoid abrasion against the vessel walls and only one face of the alloy surface was exposed to an artificial saliva medium. The metal surface was polished prior to testing to assure a consistent surface condition at the beginning of the test to decrease the heterogeneity of migration test results that might be caused by surface corrosion and passivation processes due to aging under uncontrolled environmental conditions. The results of the migration test was only slightly affected by the type of mucins used (effects of degree of mucin glycosylation were of concern) and showed metal recoveries, after a 2 hours test, close to 100% for 8 metals. Therefore, the *in vitro* methodology was able to produce consistent determinations of metal releases, with high metal recoveries, a basic requirement for a reliable estimation of mouthing exposure.

In general, good reproducibility, with coefficients of variation under 20%, was obtained for metal measurements above 10 times the method detection limit. Even more, migration results using the saliva protocol on Z45 sample, obtained by ECTX-Consultants (Hassel, Belgium), yield an inter-laboratory variability, expressed as coefficient of variation below 32% (Tony Brouwers, personal communication), suggesting a reproducible method. A more comprehensive inter-laboratory study with at least 4 laboratories would be useful to define the actual reproducibility of this method. Metal release kinetics from alloys samples was linear with time for tin in tin/lead alloy and lead in brasses; on the other hand, lead release from the whitemetal alloys and copper and zinc releases from brasses fit a first order function. For lead, a positive correlation was found for tin/lead alloys and brasses between lead released into synthetic saliva medium and total lead content of the sample. These estimates of lead release into synthetic saliva were used to predict child blood lead concentration increases using the IEUBK toxicokinetic model for lead (windows version 1.1). Only marginal blood lead increases equal to or lower than 0.2 μg/dL, were predicted for mouthing exposure of a 10 cm^2^ metallic object, using the mean daily exposure time per age group of Green 2002 [21]. Pure lead was the only sample predicted to significantly increase blood lead concentration by amounts equal to or greater than 10.2 μg/dL.

The results of this study as a whole demonstrate the feasibility of using migration data, as opposed to total metal content, to estimate exposure risk to metals in a more realistic manner. Migration data should be particularly relevant for massive samples of many materials since only the surface of an object will be exposed to biological fluids, in agreement with the more recent reviews on the subject [26].

The exposure estimates made here have inherent limitations – only limited information is available to define the frequency and duration of mouthing behaviours for children of different ages. These behaviours, and the objects which are mouthed, will likely be variable between and within countries. Similarly, IEUBK estimates of predicted blood lead impacts reflect exposure conditions and habits specific to the United States – extrapolation to other countries must be undertaken with care. The incremental increases in blood lead predicted by IEUBK are also population averages – actual blood lead increases in the individual child may be higher or lower than the mean estimates made. As noted above, inter-laboratory comparisons and validation of the proposed test method are needed. Finally, the test protocol proposed simulates metal release from gently mouthing of metallic objects. Comparison of predicted release rates with those which might actually occur has not been possible. Different rates of metal release might also occur if children exhibit more aggressive mouthing behaviors.

Whereas the focus of this study has been on the mouthing of objects, ingestion of metal-containing objects has been documented and can be of significant concern. In particular, ingestion of lead metal objects can result in acute lead intoxication and even lethality if the object does not successfully transit the gastrointestinal tract. In vitro assessment of the risk posed by lead-containing alloys would require different extraction protocols that reflect the highly acidic conditions found in the stomach [27].

## Competing interests

The authors declare that they have no competing interests.

## Authors’ contributions

PU: Chemist specialized in trace metal analysis. Carried out the metal measurements of the study and design the quality control procedures. GV: Carried out the modelling to estimate blood lead levels using IEUBK and select most appropriate estimates of mouthing time behaviour from an exhaustive bibliographic search. MO: Carried out the saliva migration test and all the previous work necessary to obtain a reproducible methodology. JA: Participate in the design, coordination, analysis and interpretation of the data and draft of the manuscript. CB: Conception and design, analysis and interpretation of the data, critical reviewing the manuscript and in the final version of the manuscript. KD: Conception and design, analysis and interpretation of the data, critical reviewing the manuscript and in the final version of the manuscript. PHR: Project management, Conception and design, analysis and interpretation of the data and draft of the manuscript. All authors read and approved the final manuscript.
